# Effect of Warm-Water Retting Pretreatment on the Physical Properties of Banana Stem and Its Fibre

**DOI:** 10.3390/ma15238462

**Published:** 2022-11-28

**Authors:** Xiangyu Yu, Yuyang Xia, Dong Liang, Wei Fu, Chenghai Yin

**Affiliations:** College of Mechanical and Electrical Engineering, Hainan University, Haiko 570100, China

**Keywords:** retting flax, banana fibre, physical characteristics, fiber extraction, characterisation

## Abstract

In this paper, warm-water flax retting was used as a pretreatment method for banana-fibre extraction. To determine the optimum conditions for flax retting, the physical properties of various parts of stems and fibres in the process of flax retting were analysed. By studying the tensile strength, elongation at break, diameter, moisture regain, and other characteristics of the fibres, the influences of bacteria and enzymes in the retting liquor on the fibre characteristics in different retting stages were determined. Through mechanical-property tests and microscopic observation of the stem skin, the change rules of the mechanical properties and degumming state of the stems were examined. The results showed that the fibre tensile strength of banana stems reached the maximum value of 45 ± 16 cN·tex^−1^ after 11 days of retting. As most resins had not been hydrolysed, fibre extraction was difficult. After 21–25 days of retting, the tensile strength of fibres was about 34 ± 10 cN·tex^−1^, elongation at break was about 1.71%, and moisture regain was about 13.56%. The fibre characteristics met the process requirements, and the tensile separation stress of the stem was small, about 0.034 MPa. This time point could be used as the optimum endpoint for retting flax in warm water, which could provide theoretical support and research basis for the recycling of banana straw. The functional groups of the extracted fibres were studied by FTIR, which confirmed the observed change rule of each component during degumming. The experimental results showed that a longer retting time corresponded with a lower content of fibre impurities, more thorough degumming, and less difficult extraction; however, strength and toughness decreased.

## 1. Introduction

Given the increasing awareness of the global energy crisis and environmental problems, the development and research of plant fibres are attracting considerable attention [1]. Plant fibres are extensively used because of their good biodegradability, renewability, thermal stability, chemical stability, and good mechanical properties [2]. The plant fibres in the stems of flax, ramie, coconut, sisal and banana meet basic textile requirements. They are usually blended with cotton and silk to make high-quality fabrics. In addition, the mechanical properties of pseudo-stem fibres are comparable to traditional reinforcements. Pseudo-stem fibres are often used as reinforcement materials. Added to the preparation of composite materials, pseudo-stem fibres can improve the mechanical properties, and enhance the thermal stability, of composite materials [3,4].

Banana fibre is a plant fibre extracted from banana-stem waste. Its light, soft, and easy-to-weave nature confers good market advantages and development potential [5]. In 2021, China’s banana-planting area was about 300,000 hectares, with an annual output of more than 1.07 billion tons. After ripe banana fruits are harvested, banana stems are processed, primarily by cutting them and transporting them to the field for free stacking. They may also be discarded on the spot and left to rot, thereby taking up land resources, causing environmental pollution, and wasting resources [6]. Therefore, to solve the environmental pollution and resource wastage caused by banana stems, appropriate fibre-extraction methods are needed. In addition to improving the physical and chemical properties of fibres, undamaged fibres can also be obtained [7].

The extraction methods of banana fibre include the mechanical separation of non-cellulose components and the chemical or microbial dissolution and decomposition of such components [8]. Mechanical extraction is efficient and avoids pollution, but the tensile strength and fineness of extracted fibres are very discrete, and the impurity content is high, which requires subsequent treatment [9,10]. The impurity content of fibre extracted by the chemical method is low, but the tensile strength and toughness of this fibre seriously declines, and it is difficult to avoid environmental pollution [11,12,13]. The difference in the physical and chemical properties of fibres extracted by the biological method is small, and the pollution in the extraction process is small. However, the fibre quality and extraction efficiency are closely related to the parameters of the biological process [14]. Huang Xiaolong et al. screened an actinomycete 51181 that could degrade banana hemicellulose in soil. This strain could be used to remove 38% of gum in banana stems [15]. Cai Yong et al. screened strains FR1 and BR2-1 from rotten banana pseudo-stems and soil samples. After 72 h of degumming, the measured gum content of banana stems decreased from 83.6% to 24.7% and 20.0%, respectively [16]. K. J. Vishnu Vardhini et al. treated banana fibres with laccase and xylanase of different concentrations and subsequently measured and analysed the physical and chemical properties of the treated fibres [17]. Kaur A et al. adopted a biological method of extracting fibres in banana stems, and then they refined the banana fibres by using xylanase and pectinase. The whiteness of the banana fibres increased by 13.27%, the brightness increased by 16.14%, and the yellowness decreased by 8.63%, compared with that before treatment, proving the effectiveness of the biological method to remove impurities in the fibres [18]. Hasan R et al. optimised the retting process. By separating and screening efficient strains, the retting period decreased from 18–21 days to 10 days, and the fibre-extraction quality improved [19].

In summary, extracting banana-stem fibre by the biological method is feasible, but it is hounded by some problems, such as high cost, low fibre-extraction efficiency, and difficult observation of the fibre-extraction process. The method of retting flax in warm water is a classical biological method. It uses the enzymes and bacteria in the banana stem to provide an environment suitable for its reproduction and then completes the fibre extraction. Under the action of the complex flora in the retting pool, the non-cellulose components in the stems are decomposed, and the fibre bundles are gradually separated from the plant tissues, thereby facilitating the extraction of the fibres [20]. The purpose of this paper was to prove the feasibility of warm water retting flax as the pretreatment process of banana stem fibre extraction, and to determine the optimum endpoint for retting flax in warm water. The following work was conducted. A bundle fibre tensile test was performed for banana-stem fibres with different lengths during retting to study the change rule of fibre tensile strength during retting. A fibre-bundle separation experiment was performed on banana-stem slices with different lengths during retting to study the change rule of fibre-bundle separation stress during retting. Microscopic observation was used to investigate the adhesion state of fibre and plant tissue at different stages to measure the difficulty of fiber extraction. Through FTIR analysis, the change rule of each component in the fiber was studied.

## 2. Materials and Methods

### 2.1. Materials

The banana stems used were from ‘Brazil Williams’ bananas planted by Danzhou agricultural community. The water source for retting flax was local tap water.

### 2.2. Pretreatment of Banana Stem Warm Water Retting

**Figure 1:**
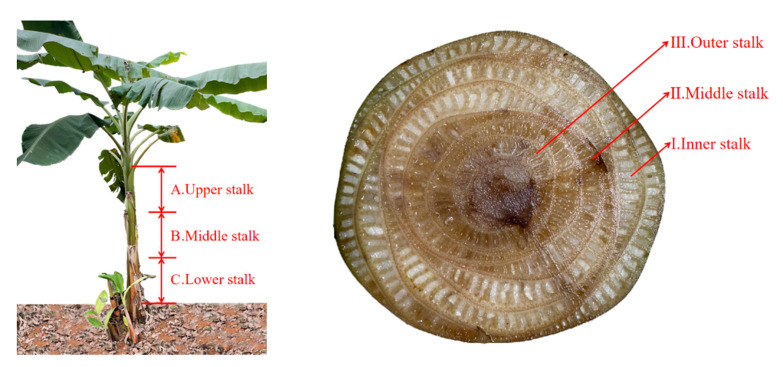
Sampling and Marking of Banana Stalks.

The banana stems were pretreated with warm water retting. Fibre is distributed in different parts of a banana tree, so the mechanical properties of fibre significantly differ [21]. As shown in Figure 1, the banana stems were divided into three equal parts from bottom to top, picked up, and placed in a retting box with the upper stem marked A, the middle stem marked B, and the lower stem marked C. The inner stem was marked as I, the middle stem as II, and the outer stem as III. Water was injected according to a bath ratio of 1:20 (mass ratio) [22]. Flax retting was conducted with warm water at natural temperature (32–38 °C) and humidity (70% relative humidity), and the start date of flax retting was recorded.

### 2.3. Bundle Tensile Test

According to the ASTM D3379-75 standard, the tensile strength and elongation at break of fibre bundles were measured using a tensile tester (Instron 3343, 2580 Series Static Load Cells, measuring range 2.5–500 N, Canton, UK). The banana stems were stripped and sampled manually every other day from the fifth day of retting. The sampling parts were evenly distributed throughout the entire stem slice. Twenty fibre samples were taken from each stem slice to obtain banana stem fibre bundle samples with a length of more than 40 mm. The fibre bundles were clamped between two pneumatic clamps with a test length of 25 mm, and the traveling speed of the pneumatic clamp was set to 2 mm/min.

### 2.4. Determination of Fibre Diameter of Banana Stem

Ten fibres were randomly taken from each stem slice, and six different positions were observed for each fiber. The diameter of fibres was measured with a polarised light microscope (Nikon LV100POL, Tokyo, Japan) at 100× magnification, and the average value and coefficient of variation were calculated.

### 2.5. Determination of Moisture Regain of Banana-Stem Fibre

The determination of moisture regain of banana-stem fibres was in accordance with the GB6503-2008 standard, using a dryer (Lichen 101-0BS, Shanghai, China) and a one ten-thousandth balance (DEJUINSPR 124ZH/E). Five samples with a length of 40 mm for each group, were weighed, dried at 105 °C for 2 h, and the dried mass then weighed. Three groups of tests were conducted for each batch of samples, and the moisture regain of the samples and the average value were calculated.

(1) $$\mathrm{moisture}\;\mathrm{regain}=\frac{\mathrm{wet}\;\mathrm{weight}-\mathrm{dry}\;\mathrm{weight}}{\mathrm{dry}\;\mathrm{weight}}\times 100\%$$

### 2.6. Banana Stem Tensile Separation Test

**Figure 2:**
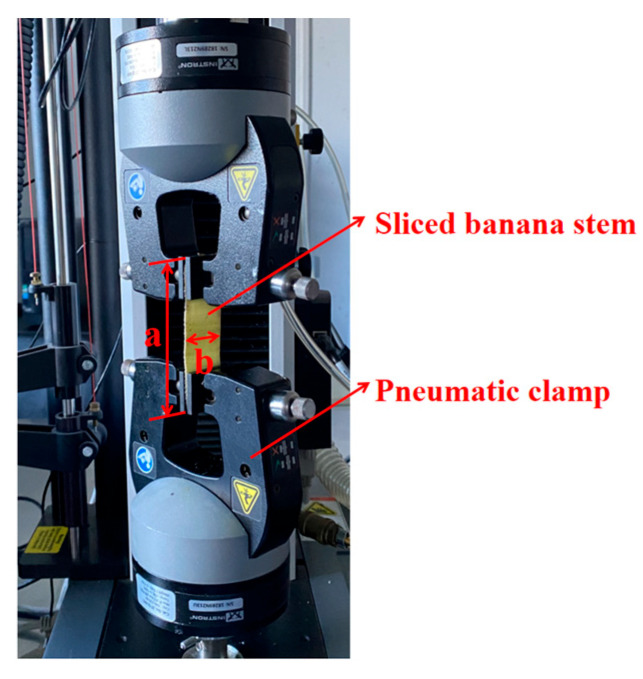
Straw tensile test.

The retting time and different parts of the sample are the main factors affecting the tensile separation stress of a fibre bundle. From the beginning of retting, the banana stem was sampled every other day. A blade was used to separate the outer skin of the banana stem, and the sample was cut into rectangular samples with a length of a = 60 mm and a width of b = 30 mm. As shown in Figure 2, three samples were collected from each group to record the thickness, d, of the sample, and a tensile testing machine (Instron 3343, measuring range = 2.5–500 N, Canton, UK) was used to measure the ultimate tensile force of the tensile separation of the stem. The sample clamping direction was perpendicular to the fibre direction, and the sample end was parallel to the clamp end. The test length was 30 mm. The travel speed of the pneumatic clamp was set to 2 mm/min until no significant change occurred in tensile force. The data and stress diagram were derived to calculate the tensile separation limit stress of the stem.

(2) $$\sigma t=\frac{F_{\max}}{S}$$

S = B × d(3)

where σt is tensile strength, F_max_ is ultimate tension, and S is fracture section area.

### 2.7. Statistical Methods and Tools

This paper mainly used the descriptive statistical analysis method. The specific data statistical analysis process was as follows. In the mechanical property analysis experiment, the data corresponding to the mechanical characteristic curve of the sample was imported into Microsoft Office Excel 2013 software, and the tensile starting point (the first data point with a load greater than 0.2 N) and the maximum load point were selected. The data was imported into Origin 2017 software to draw the statistical graph. In the determination of other physical properties, the data measured in the test were counted with Statistical Product and Service Solutions 19.0 software to complete the calculation of standard deviation, average value and coefficient of variation.

### 2.8. Microscopic Observation on the Outer Skin of Stem Slice

The surface of the outer epidermis of the stem slice was observed under an inverted microscope (Leica DMIL LED, Wetzlar, Germany), and the magnification was 40 times.

### 2.9. FTIR Spectroscopy Analysis

The change in functional groups of the samples could be evaluated by FTIR spectroscopy. The instrument used was a near-infrared spectrometer (BRUKER T27, Karlsruhe, Germany). Initially, samples of banana-stem fibres that had been retted for 5, 15, and 25 days were initially ground and mixed with KBr. The mixture was ground in an agate mortar and compressed into discs. The spectral recording range of the sample was 600–4000 cm^−1^, and the resolution was 2 cm^−1^. The sample was scanned 32 times, and the functional image with wavenumber as independent variable was the output.

## 3. Discussion of Results

### 3.1. Bundle Tensile Test

#### 3.1.1. Tensile Strength of Bundle Fibre

**Figure 3:**
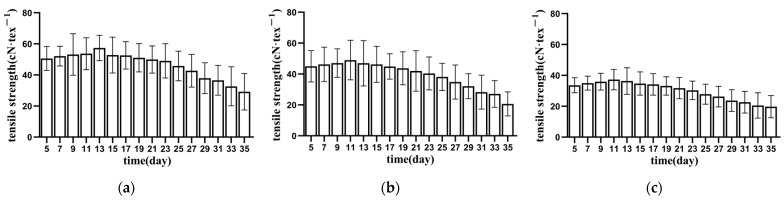
Tensile strength of inner banana stem fibres. (**a**) AⅠ; (**b**) AⅡ; (**c**) AⅢ.

**Figure 4:**
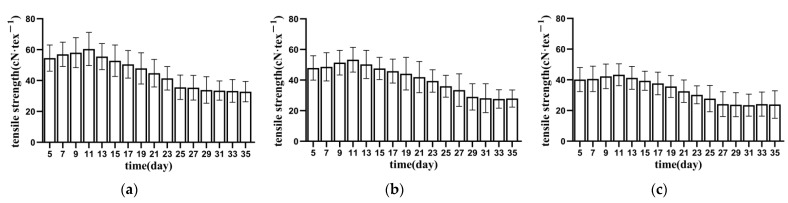
Tensile strength of middle banana stem fibres. (**a**) BⅠ; (**b**) BⅡ; (**c**) BⅢ.

**Figure 5:**
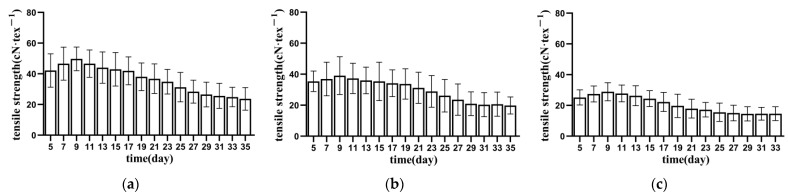
Tensile strength of lower banana stem fibres. (**a**) CⅠ; (**b**) CⅡ; (**c**) CⅢ.

Figure 3, Figure 4 and Figure 5 show the relationship between the tensile strength of the fibres in each part of the banana stem and retting time. The tensile strength of the fibres in the banana stem increased slowly during the 5–9 days of retting. The changes in this process included the dissolution of pigments and soluble substances in the fibres and the decomposition of the gum on the fibre surface (primarily owing to the decomposition of the aerobic pectin-decomposing bacteria and the consumption of oxygen in the water), and the linear density and fineness of the fibres decreased slowly. Consequently, the tensile strength of the fibres indirectly increased. After retting for 9–11 days, pectin rapidly hydrolysed, and fiber linear density and fineness rapidly reduced. The tensile strength of the fibres significantly improved, reaching the maximum value of 45 ± 16 cN·tex^−1^ for the entire retting process at about 11 days (about 15% higher than that of untreated fibres). The changes in this process included the decomposition of some pectin in the fibres (primarily due to the role of anaerobic pectin-decomposing bacteria) and the direct or indirect dissolution of lignin and its hemicellulose connected with cellulose by the action of enzymes [23]. After retting for 11–31 days, fibre tensile strength continued to decrease, and the whiteness gradually decreased. The changes in this process included the hydrolysis of cellulose, lignin, hemicellulose, and deep pectin, primarily caused by the rapid propagation of anaerobic pectin-decomposing bacteria, the decomposition of pectin, and the gradual exposure of hemicellulose and cellulose, which induced microorganisms to secrete xylanase and cellulose [24]. In turn, hemicellulose and cellulose degradation increased the contact area between pectinase and pectin substances and promoted the decomposition of pectin substances. However, the content of cellulase increased and cellulose continued to hydrolyse, leading to a decline in fibre strength and toughness [25]. After 31 days of retting, although the fibre strength did not decrease significantly (about 60% of the non-retted fibre), diffuse mildew gradually appeared, and the fibre whiteness decreased rapidly. Accordingly, the retting test was stopped on the 35th day. As there is no unified standard for the tensile strength of banana fiber, by referring to the relevant standards of ramie, the tensile strength of the fiber is required to be higher than 30 cN·tex^−1^. It is difficult for the lower stem to reach this strength standard, and so it is often not used as the raw material for fiber extraction in production [26]. In comprehensive consideration, the end time of retting should be earlier than 25 days.

By comparing the tensile-strength changes of the fibres in the upper, middle, and lower parts of each layer of culm, we found that the strength of banana stem fiber has the same distribution law as hemp fiber, that is, the lower fiber has the lowest strength and the middle fiber has the highest strength. The reason for this is that the lower fiber diameter is significantly higher than the upper and middle fiber diameters. The larger the fiber diameter, the higher the probability of containing defects. The difference between the middle fiber diameter and the upper fiber diameter is small, but the proportion of cellulose in the middle fiber is higher than the upper fiber, so the fiber strength is higher [27]. Under the same external conditions, the lower fibre was retted faster than the upper and middle parts, and the retting of flax was more thorough. The reason for this phenomenon was that the lower stem was close to the soil where it grew, and it carried more enzymes and bacteria required for the retting process, leading to faster propagation of enzymes and bacteria and faster stem corruption [28].

The tensile mechanical properties of fibre bundles are scattered, affected by various parameters, such as sampling position, growth characteristics, and degumming [29]. Through sufficient repeated groups and multipoint fibre random sampling, the true change rule of tensile strength of fibres in each part of the stem slice during processing was obtained, but the dispersion of data increased to a certain extent. Figure 3, Figure 4 and Figure 5 show that the dispersion of tensile strength of each group of fibres initially increased and then decreased. The increase was due to different oxygen contents in each area and the activity and reproduction speed of pectin bacteria caused by the reproduction of aerobic pectin-decomposing bacteria after the stem slices were stacked and soaked. Secondly, retting straw aggravates the defects in some fibres, making the fibres break ahead of time at the defects [30]. However, with the retting of stem slices, the retting water intruded into the stem slices, the water-bath environment tended to be consistent, and the concentrations of bacteria and enzymes tended to be consistent, so the degree of dispersion decreased.

The tensile mechanical properties of fibres are the most important physical properties of fibres. In order to obtain high-quality fibres, the higher the tensile strength of fibres, the better, and the smaller the discrete type of fiber tensile strength, the better. It is comprehensively considered that 21–25 days is the best time end point for retting.

#### 3.1.2. Breaking Elongation of Bundle Fibre

**Figure 6:**
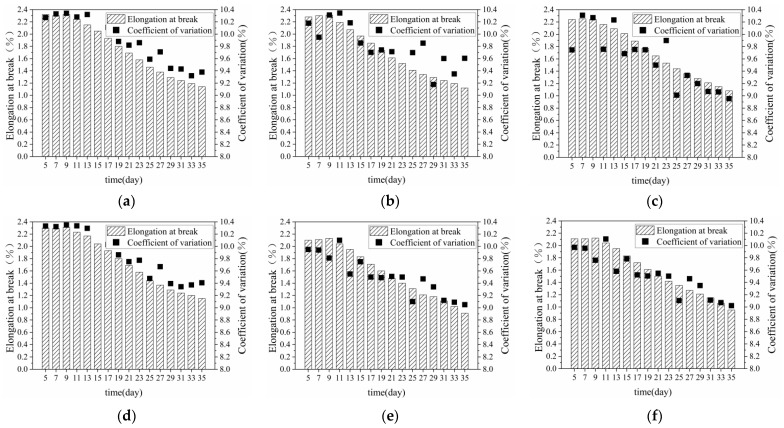

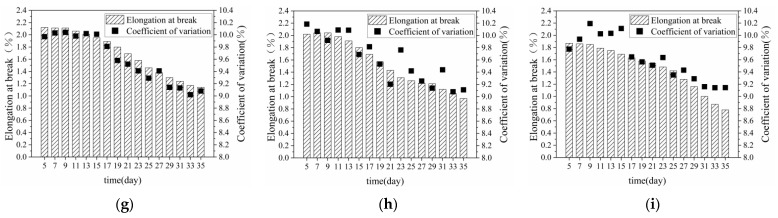
Breaking elongation of fibres in different parts of banana stalks (**a**) AⅠ; (**b**) AⅡ; (**c**) AⅢ; (**d**) BⅠ; (**e**) BⅡ; (**f**) BⅢ; (**g**) CⅠ; (**h**) CⅡ; (**i**) CⅢ.

Figure 6 shows the elongation at break of banana-stem fibres after retting treatment. The elongation at break of the fiber exhibited a downward trend, as a whole, in the retting process, from 2.1 ± 0.2% in 5 days to 1.3 ± 0.2% in 25 days and 1.0 ± 0.2% in 35 days. The elongation at break reflected the elasticity and toughness of the fibre, and was primarily affected by two aspects; the chemical components in the fibre, which were the main influencing factors, and hemicellulose, which endowed the fibre with elasticity and toughness [31]. Throughout the entire retting process, hemicellulose was always decomposed by the action of xylanase, and the content of hemicellulose, fibre toughness, and elongation at break were reduced. The fibre fineness was the secondary influencing factor, and when it decreased, the fibre toughness decreased, and the fibre elongation at break decreased. The coefficient of variation of fibre elongation at break reflected the dispersion degree of fibre elongation at break, which was primarily affected by the distribution difference of enzymes and bacteria in various parts of the stem during retting. The enzyme and bacteria in the retting pool gradually changed from local rapid propagation to overall average, and the variation coefficient of fibre elongation at break initially increased and then decreased. After the fiber in banana stems was treated with warm water retting, the dispersion of fiber breaking elongation decreased. The coefficient of variation decreased by about 0.8% at 25 days of retting, and the coefficient of variation decreased by about 1% at 35 days of retting. The retting reduced the difference in elasticity between samples of the same group.

### 3.2. Effect of Retting Flax on Fibre Diameter

**Figure 7:**
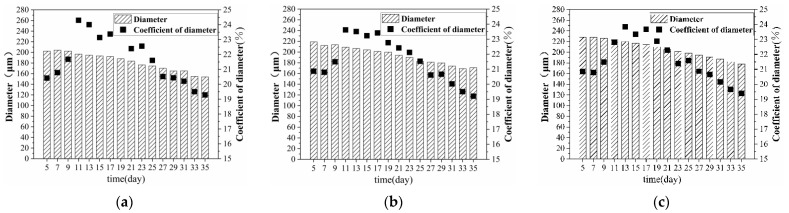

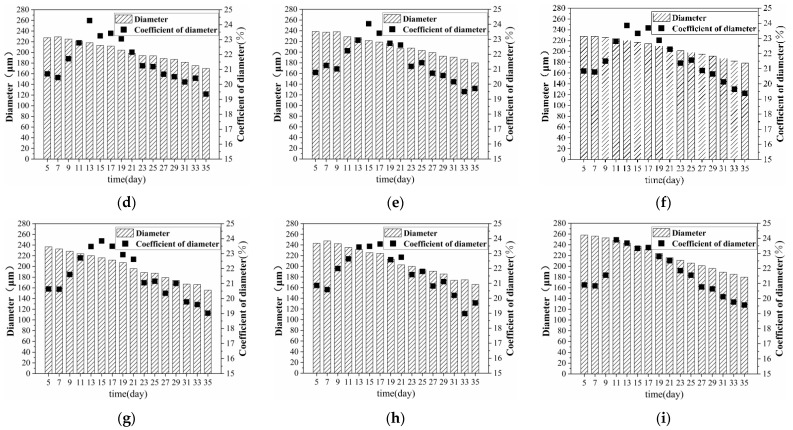
Diameter of fibre in different parts of banana stem (**a**) AⅠ; (**b**) AⅡ; (**c**) AⅢ; (**d**) BⅠ; (**e**) BⅡ; (**f**) BⅢ; (**g**) CⅠ; (**h**) CⅡ; (**i**) CⅢ.

Figure 7 shows that the fibre diameter decreased with retting treatment, which was due to the gum, hemicellulose, lignin, cellulose, and other components in the fibre becoming hydrolysed and dissolved by enzymes and bacteria. A higher removal rate corresponded with a smaller fibre diameter. In the whole retting stage, the fibre diameter changed by about 56 µm. After the ninth day of straw retting, the diameter decreased significantly, which corresponded with the strong fermentation stage, and the straw quickly decayed. Notably, the fibre diameter of the lower stem was the highest owing to plant-growth characteristics, but after retting under the same conditions, the fibre diameter was lower than that of the middle stem. The reason was that the difference in the number of bacteria and enzymes in the stem caused the lower stem to decay more quickly and thoroughly. The coefficient of variation reflected the degree of dispersion of the data. The coefficient of variation of fibre diameter was high (about 19.4–23.9%), but considered to be valid, consistent with earlier studies [32]. This finding was due to the obviously different fibre diameters of different sampling parts in a single stem, and different parts of a single fibre showed different diameters [33]. The coefficient of variation of diameter indicated that the dispersion degree of fibre diameter initially increased and then decreased with the retting process, which was also a process in which the distribution of enzymes and bacteria in the retting pool tended to be uniform.

### 3.3. Effect of Retting Flax on Fibre-Moisture Regain

As shown in Table 1, Table 2 and Table 3, the fibres in different stages of retting showed different moisture regain, which decreased from 15.46% to 12.27%. The moisture regain of fibre reflects the ability of the fibre to absorb and store water. The main components of fibre are hemicellulose, cellulose, and lignin, which have different water-absorption abilities. Among them, hemicellulose has the strongest water-absorption ability, followed by cellulose, and lignin has the weakest [34]. After retting, the content of hemicellulose and lignin in the stems decreased, but the dissolution of hemicellulose played a more important role [35], enabling continued decrease of the moisture regain of fibres. The moisture regain of the fibre in the retted lower stem was significantly higher than that in the upper and middle parts, which might be due to the dissolution of deep colloid and hemicellulose causing the fibre in the lower stem to have a more open structure and be able to accommodate more water molecules [36].

From the standard deviation of the moisture regain of banana stem fibre, we found that the moisture regain was a very stable physical property of banana fiber. Its standard deviation was always lower than 5% of the average value, and this balanced property was not affected by the degumming in warm water. This property means banana fibres have more application scenarios [37].

### 3.4. Separation of Fibre and Plant Tissue

#### 3.4.1. Straw Tensile Separation Test

**Figure 8:**
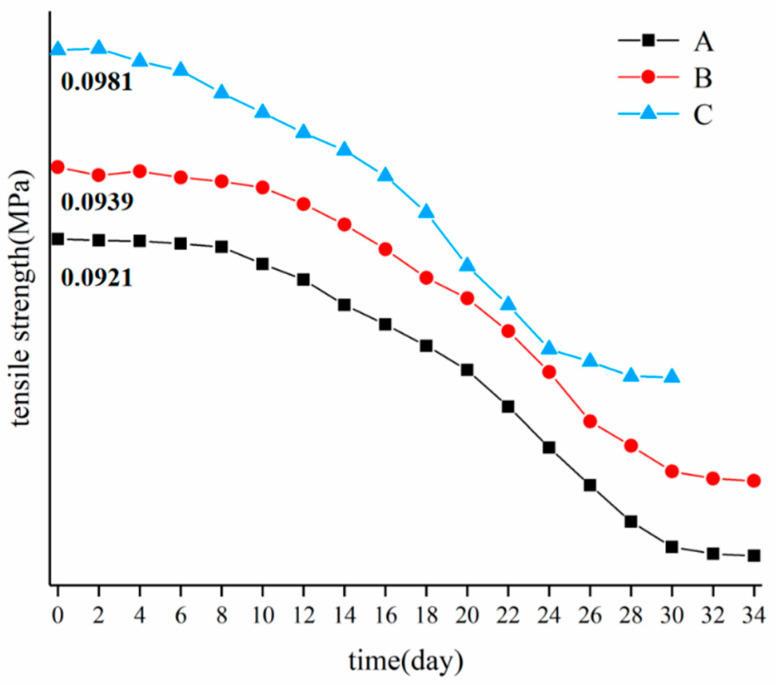
Tensile strength of banana stem sheath.

Figure 8 shows the tensile-strength diagram of the outer skin of the stem. As shown in the figure, the maximum stress of the stem before breaking changed with the retting time, the stem primarily went through a physical process in about 0–6 days of retting, the stem absorbed water and expanded, and the pigment and soluble substances dissolved, and the tensile strength had no significant change (about 0.095 MPa). About 6–16 days after retting, pectin in the stem was decomposed by pectin bacteria, and xylanase was released at the same time, leading to the dissolution of lignin connected with hemicellulose, and the tensile strength of the stem was reduced. At about 16–28 days, with the continuous accumulation of pectinase in the retting process, the dissolution rate of pectin increased, and the tensile strength of the stem decreased rapidly, reaching the minimum of 0.0165 MPa (decreased by about 80%) about 30 days after retting. By analysing the change in tensile strength of the outer culm, we found that the tensile strength of the lower culm began to decrease earlier than that of the upper and middle parts. The reason for this was the same as that in the fibre bundle tensile test, that is, the different distribution of bacterial colonies in different parts of banana culm.

#### 3.4.2. Microscopic Observation on the Outer Epidermis of Stem Slices

**Figure 9:**
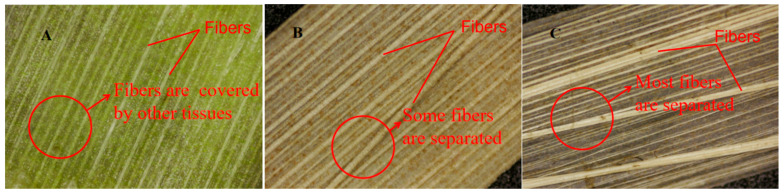
Microscopic image of banana stem outer skin (**A**) 5 days of retting with warm water; (**B**) 15 days of retting with warm water; (**C**) 25 days of retting with warm water.

The microscopic images of the outer skin of banana-stem slices after 5, 15, and 25 days of retting are shown in Figure 9. As shown in Figure 9A, banana stems had no obvious corrosion after 5 days of retting. The outer skin of the stems was difficult to peel. Fibres were visible and tightly wrapped with plant tissue. At this time, fibre extraction was difficult, and the fibres broke easily. Figure 9B shows that banana stems severely corroded after 15 days of retting, and all fibres were visible. Some fibres left the skin after being soaked in water. At this time, the difficulty of fibre extraction was moderate, and the fibre strength and toughness were good. Figure 9C shows that after 25 days of retting, banana stems were completely corroded, the outer skin naturally separated from plant tissues, and some fibres naturally separated from the outer skin. All fibres were visible, and most fibres were separated from the skin after soaking in water. At this time, fibre extraction was difficult, fibre strength was moderate, but fibre toughness was poor.

### 3.5. FTIR Spectral Analysis

**Figure 10:**
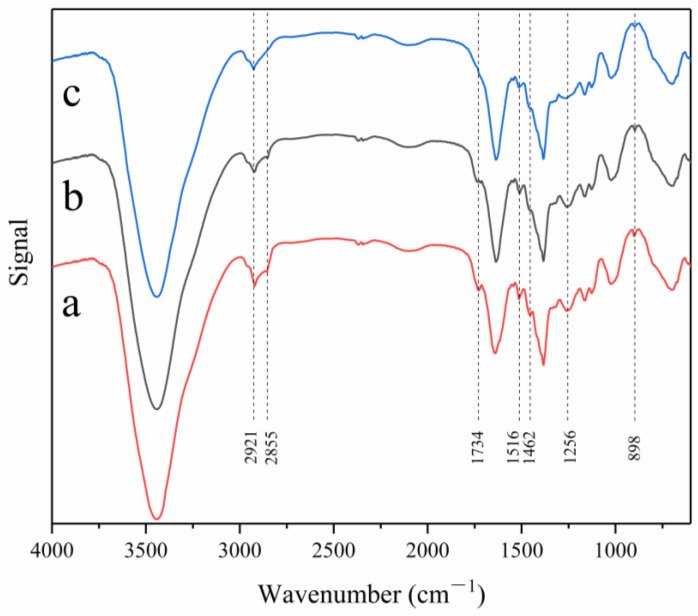
Spectrum of banana fibre (**a**) 5 days of retting with warm water; (**b**) 15 days of retting with warm water; (**c**) 25 days of retting with warm water.

Figure 10 shows the FTIR spectra of C III fibre after 5 days of retting (a), 15 days of retting (b), and 25 days of retting (c). Each component in the fibre had different chemical compositions, and the functional groups also varied. Through the Fourier transform near-infrared spectroscopy analysis of the fibres, the change in chemical composition of the fibres induced a change in the peaks of these functional groups in the FTIR spectrum, which facilitated study of the change rule of each component in the fibre at different stages of retting. The comparison of corresponding peak positions of each group in the fibres is provided in Table 4. A peak value at 3440 cm^−1^ could be reached, corresponding with OH stretching vibration [38]. The two peaks at 2921 and 2855 cm^−1^ corresponded with the CH tensile vibration of CH and CH2 in cellulose and hemicellulose, respectively [39]. The peak at 1734 cm^−1^ corresponded with the CH tensile vibration in hemicellulose [40], that at 1516 cm^−1^ corresponded with the C=C tensile vibration of aromatic group [41], that at 1462 cm^−1^ corresponded with the bending vibration of lignin CH2 [42], that at 1256 cm^−1^ corresponded with the C-O tensile vibration of the acetyl group or the C-O-C tensile vibration of the ester group [43], and that at peak at 898 cm^−1^ corresponded with the glucocyclic β-glycosidic bond [44].

Through FTIR analysis, we concluded that no new functional groups were introduced into the fibres during retting, so no other impurities were introduced into the retting process. By comparing curves, a, b, and c, in Figure 10, at 3440, 2921, and 2855 cm^−1^, the peak value gradually increased as retting proceeded, indicating that hemicellulose and lignin continuously hydrolysed during retting. With the gradual weakening of the intensity of the peak in the process of retting at 1734 and 1256 cm^−1^, the content of hemicellulose gradually decreased, the peak at 1734 cm^−1^ in curve c was missing, and the peak at 1256 cm^−1^ significantly weakened, indicating that the content of hemicellulose in banana fibres was very small after 25 days of retting. With the treatment of fibres at 1516 and 1256 cm^−1^, the peak gradually weakened because of the hydrolysis and dissolution of lignin, and the lignin content in the fibres decreased. The characteristic peak at 898 cm^−1^ existed in all curves, corresponding with the β-glycoside bond representing cellulose, indicating that cellulose was well retained throughput the entire retting process.

## 4. Conclusions

Retting flax in warm water under natural conditions was used to pretreat banana stems, and the effects of retting treatment on the physical and chemical properties of banana stems and banana fibres from different parts were studied. Bacteria, such as pectin-decomposing bacteria, and enzymes, such as xylanase and pectinase existing in the banana stem, multiplied in the retting pool. These enzymes and bacteria changed the chemical composition of fibres, and the content of hemicellulose, lignin, and pectin gradually decreased, and the mechanical properties, diameter and moisture absorption of the fibers changed. Experimental results showed that the tensile strength of the fibers increased slowly at first and then decreased rapidly with the retting process. The maximum tensile strength, 45 ± 16 cN·tex^−1^, was reached after 11 days of retting, and was lower than the tensile strength standard of high-quality fiber after 25 days of retting, and reached the minimum tensile strength of 19 ± 5 cN·tex^−1^ after 31 days of retting. The elongation at break of the fibres remained unchanged at first and then decreased. At 9 days after warm water retting, it remained at 2.1 ± 0.2% and then decreased to 0.9 ± 0.1%. The fiber diameter decreased from 240 ± 20 μm to 187 ± 7 μm. The moisture absorption of the fiber was usually reflected by the moisture return rate, which decreased from 15.0 ± 0.9% to 12.6 ± 0.8%. The above physical properties became concentrated. A longer retting time corresponded with a lower content of fibre impurities, more thorough degumming, and less difficult extraction; however, strength and toughness decreased. According to comprehensive analysis, in order to obtain high-quality fibres with tensile strength up to standard and concentrated physical properties under the condition of low fiber extraction difficulty, the end time of retting should be about 21–25 days. Notably, the lower stem carried more enzymes and bacteria required for retting, so the biological reaction process was more rapid and thorough. If centralised retting was performed, retting should be properly terminated ahead of time based on the time endpoint determined in this paper. Finally, the removal of hemicellulose and lignin and the retention of cellulose were confirmed by FTIR spectroscopy analysis of banana fibres. No chemical reagents were used throughout the entire retting process, so the cellulose was not seriously damaged. The retting process was environmentally friendly, and almost no pollutants were generated.

## Figures and Tables

**Table 1:** Retting duration and fibre moisture regain of inner stem.

Position		AI		BI		CI	
	Nature	Regain	REGAIN—Standard Deviation	Regain	Regain—Standard Deviation	Regain	Regain—Standard Deviation
Time (Day)							
5		15.92	0.64	15.25	0.61	14.28	0.57
7		15.84	0.64	15.30	0.60	14.42	0.58
9		16.07	0.65	15.28	0.61	14.31	0.58
11		15.84	0.64	15.14	0.60	14.25	0.57
13		15.72	0.63	14.87	0.59	14.09	0.55
15		15.44	0.63	14.56	0.58	14.15	0.57
17		15.28	0.61	14.26	0.58	14.14	0.57
19		14.80	0.60	13.91	0.56	13.86	0.55
21		14.58	0.59	13.61	0.55	13.66	0.56
23		14.42	0.60	13.32	0.53	13.35	0.53
25		13.93	0.59	13.01	0.52	13.04	0.52
27		13.63	0.58	13.00	0.53	12.81	0.51
29		13.42	0.58	13.06	0.52	12.58	0.49
31		13.18	0.58	12.98	0.51	12.25	0.50
33		12.85	0.53	13.04	0.53	11.99	0.48
35		12.72	0.53	13.05	0.51	11.82	0.46

**Table 2:** Retting duration and fibre moisture regain of middle stem.

Position		AII		BII		CII	
	Nature	Regain	Regain—Standard Deviation	Regain	Regain—Standard Deviation	Regain	Regain—Standard Deviation
Time (Day)							
5		15.83	0.63	15.25	0.61	14.17	0.58
7		15.76	0.63	15.30	0.62	14.30	0.56
9		15.96	0.65	15.28	0.61	14.20	0.58
11		15.72	0.63	15.14	0.60	14.13	0.55
13		15.59	0.62	14.87	0.59	14.00	0.56
15		15.31	0.62	14.56	0.58	14.01	0.56
17		15.16	0.61	14.26	0.57	13.75	0.53
19		14.70	0.59	13.91	0.57	13.99	0.57
21		14.44	0.57	13.61	0.54	13.76	0.55
23		14.22	0.55	13.32	0.53	13.73	0.55
25		13.74	0.55	13.01	0.52	13.53	0.53
27		13.14	0.53	13.00	0.51	13.44	0.54
29		12.40	0.50	13.06	0.52	13.24	0.53
31		12.07	0.48	12.98	0.53	13.23	0.52
33		12.17	0.50	13.04	0.52	13.22	0.53
35		12.13	0.50	13.05	0.51	13.36	0.53

**Table 3:** Retting duration and fibre moisture regain of outer stem.

Position		AⅢ		BⅢ		CⅢ	
	Nature	Regain	Regain—Standard Deviation	Regain	Regain—Standard Deviation	Regain	Regain—Standard Deviation
Time (Day)							
5		15.47	0.63	14.89	0.60	13.94	0.57
7		15.51	0.61	14.91	0.62	14.02	0.56
9		15.54	0.63	14.92	0.61	13.93	0.55
11		15.56	0.62	14.71	0.59	13.88	0.56
13		15.31	0.61	14.49	0.58	13.81	0.55
15		15.09	0.61	14.12	0.55	13.71	0.55
17		14.84	0.58	13.83	0.55	13.60	0.53
19		14.48	0.58	13.52	0.56	13.55	0.55
21		14.20	0.57	13.21	0.53	13.51	0.54
23		13.81	0.56	12.92	0.52	13.38	0.54
25		13.36	0.53	12.61	0.51	13.25	0.55
27		12.89	0.51	12.59	0.50	13.11	0.51
29		12.12	0.48	12.61	0.49	12.99	0.52
31		11.85	0.48	12.62	0.50	12.92	0.52
33		11.84	0.49	12.61	0.51	12.86	0.52
35		11.86	0.46	12.60	0.50	12.88	0.50

**Table 4:** Corresponding peak positions and sources of functional groups in fibres.

Functional Group	Vibration Form	Wave Number (cm^−1^)	Source
OH	*V*_OH_	3100–3800	cellulose, lignin, hemicellulose
CH_2_	*V* ^as^_CH_	2921–2931	cellulose, hemicellulose
CH	*V* ^s^_CH_	2855–2895	cellulose, hemicellulose
COOR	*V*_C=O_	1750–1730	hemicellulose
C=C	*V* ^as^_C=C_	1650–1450	lignin
CH_2_	*δ*_CH_	1445–1485	lignin
C-OH	*V*_C-O_	1200–1300	hemicellulose
COOR	*V*_C-O-C_	1000–1300	lignin
Glucose ring	β- Glycosidic bond	898	cellulose

## Data Availability

The data that support the findings of this study are available upon request from the authors.
